# First insights into molecular basis identification of 16 s ribosomal RNA gene of *Staphylococcus aureus* isolated from Sudan

**DOI:** 10.1186/s13104-021-05569-w

**Published:** 2021-06-25

**Authors:** Manal A. Gumaa, Abeer Babiker Idris, N. E. Bilal, Mohamed A. Hassan

**Affiliations:** 1 Department of Medical Microbiology, Faculty of Medical Laboratory Sciences, University of Khartoum, Khartoum, Sudan; 2 Department of Medical Microbiology, Faculty of Medical Laboratory Sciences and the Director of Central Research Laboratory, University of Khartoum, Khartoum, Sudan; 3 Department of Bioinformatics, DETAGEN Genetic Diagnostics Center, Kayseri, Turkey

**Keywords:** *Staphylococcus aureus*, 16S rRNA gene, Phylogenetic tree

## Abstract

**Objective:**

In this study, we analyzed the molecular evolution of *Staphylococcus aureus* isolates using 16S rRNA gene and phylogenetic analysis to detect the prevalence of *S. aureus* infections in Sudan.

**Results:**

Molecular detection of *S. aureus* has shown that 20 (43.47%) of patients were positive for *S. aureus*. The phylogenetic tree of 16S rRNA sequences was divided into three lineages of *S. aureus* isolates detected from wound infections in Sudan. Nucleotides base-pair substitution was appeared at position 249. This mutation do not linked with Macrolides, Lincosamides and Streptogramines b resistant phenotype. Further studies should investigate the effect of that mutation on resistance to other antibiotics.

**Supplementary Information:**

The online version contains supplementary material available at 10.1186/s13104-021-05569-w.

## Introduction

The increasing number of multidrug-resistant (MDR) staphylococcal infections has created the need to investigating basic questions about how genetic variations that cause antibiotic resistance evolved within the population of bacteria [1–3]. Consequently, in recent years, sequence analysis of the 16S rRNA gene is becoming more common as a genetic marker to confirm our understanding of *Staphylococcus aureus* phylogeny and taxonomy and increasingly prevalent in the clinical environment [4, 5].

In fact, the geographic linkage within *S. aureus* is likely a result of interfamilial transmission incorporated with rearrangement within local communities [6, 7]. Few studies have been published in Sudan on 16S rRNA gene sequencing. As described by Hassan et al. [8], who showed the significance of microbial identification and phylogenetic markers for Staphylococci species from Sudanese isolates used for taxonomy. Prior research also been explored in Sudan by Merghani et al., suggested that PCR assay with primers targeted to the 16S rRNA gene sequence offered a useful method for the identification of bacteria to the species level and differentiated one species from others [9, 10]. However, these studies cannot be considered as conclusive because the results did not correspond to the results of polyphasic taxonomy, and they found the related species cannot always be distinguished from each other [11].

Therefore, another promising line of research would be to detect the presence of mutations in 16S rRNA and investigate the conservation of *S. aureus* sequences. These points have never previously been addressed in Sudan and the information regarding the African population is limited. This paper addresses to understand more completely the key tenets about molecular analysis of the 16 rRNA gene and phylogeny approach of *staphylococcus aureus* strains isolated from Sudanese patients with wound infections [12].

## Main text

### Materials and methods

#### Clinical isolates

The study was carried out at Tow hospitals in Khartoum state: Soba University Hospital which started working in 1975 and affiliated to the University of Khartoum as the main training center for the medical students. It is affording different medical specialties and gives high grade diagnostic services through specialized units. The hospital receives patients from all states of Sudan with 500 beds capacity and the surgery departments have two general surgical units. The other is Military Hospital which present in Omdurman city and established at 1958. It represented in a complex of seven specialized hospitals totalizing 722 beds and 8 ICUs which treated patients from different regions of Sudan.

The process was accomplished in Medical Laboratory College at Khartoum University, Department of Microbiology and Molecular Biology. The study population includes patients who attended to hospitals for wound infections or post-operative surgical site infections. A questionnaire was used to collect personal and demographic data, the clinicobiologic data was collected from patient’s medical record in the selected hospitals which included age, gender, hospital unit, in/ out patient and the site of infection. Forty-six wound swabs which collected was anonymized and de-identified prior to analysis.

#### Bacterial identification

After the samples were received to the laboratory, they inoculated on Mannitol salt agar (Oxoid CM0085B), each isolated strains of Gram positive cocci were sub cultured on nutrient agar (Oxoid CM0 003B) and incubated at 37 ºC over night for biochemical reactions [13]. *S. aureus* were identified by fermentation of mannitol, colonial morphology, Gram stain, Catalase test and coagulase test using conventional methods [14].

#### Molecular characterization

##### PCR Amplification of 16S rRNA Gene

The genomic DNA of *S. aureus* isolates were extracted from nutrient agar plates by guanidine chloride method as described previously by Alsadig et al. [15]. Then, PCR was carried out using universal oligonucleotide primers: 27F (5′-AGAGTTTGATCCTGGCTCAG-3′) And 1492R (5′ TACGGTTACCTTGTTACGACTT-3′) [16]. The reaction mixture was included 1 μl of bacterial DNA, 22 μl of δdH2O, and l μl each primer in a final reaction volume of 25 μl. This mixture was added to the PCR master mix (GoTaq, Promega, USA) following the manufacturing guide. Then, run with a thermal cycler (SensoQuest, Germany) as follows: 30 cycles were performed in a thermocycler, each cycle has three steps of denaturation (95 °C for 1 min), annealing (54 °C for 1 min), extension (72° C for 3 min) and final extension time of 72 °C for 5 min [8]. Amplified products were analyzed by conventional electrophoresis, Bands were determined using an Imagemaster VDS image analysis system (SCIE-PIAS VISION U.K) [17]. The Sizes of the amplified products using universal primer were 1500 bp which suggesting that bands is 16S rRNA gene.

##### Sequencing of 16S rRNA gene

DNA purification and Standard Sanger sequencing was conducted for ten isolates which were packaged according to the National Health Research Ethics Committee authorization and following the instructions of the sequencing company (Macrogen Inc. Seoul, Korea). The sequences was submitted in NCBI: https://www.ncbi.nlm.nih.gov, with the accession numbers: from MT154222 to MT154231.

##### Bioinformatic analysis

Nucleotide sequence isolates were visualized using Finch TV program (version 1.4.0) [18]. In order to search for nucleotide sequences similarity, Genbank databases were used by online program nucleotide BLAST (http://www.ncbi.nlm.nih.gov/blast/Blast.cgi) [19]. Closely related sequences were explored from NCBI and subjected to multiple sequence alignment by BioEdit program (version 7.2) [20, 21]. Gblocks was used to estimate the quality of each sequence, edit and eliminate poor quality sequences [22]. The Neighbor-Joining phylogenetic tree was carried out by MEGA software using default parameters (http://www.megasoftware.net/index.html) [23–25].

### Results

#### Analysis of 16S rRNA sequences

10 sequences of 16S rRNA of *S. aureus* from Sudanese patients were characterized by PCR to investigate the mutations and their conservative nature; Products band had a clear chromatogram. Sequence analysis by BLASTn revealed similarity with few differences with *S. aureus* from Japan (LC508802), China (MN923027), Pakistan (MN611106), Nigeria (MN606199), Bangladesh (MN611246), Egypt (MN556575), Iraq (MN555444) and China (MN652637) as shown in Additional file 1: Table S1. Regarding mutations, the alignment of our isolated sequences showed that tow isolates (21 and 38) exhibited base-pair substitution was appeared at position 249 from A to G, Additional file 1: Figure S1(A). Multiple sequence alignment of the isolates with *S. aureus* Genbank strains confirmed the presence of that variation from isolates and from selected published nucleotide sequences compared with the reference strain, Additional file 1: Figure: S1(B).

#### Structuring of the Phylogenetic tree (maximum likelihood tree)

The cladogram graphic of a phylogenetic tree diverged into three lineages. All the Sudanese *S. aureus* strains clustered with strains from different countries. The 16S rRNA sequence of strains 7 and 38, although clustered with other global strains in one clade, had a novel A→G substitution at nucleotide position 249 as a kind of strain evolution. However, mutant strains 7 and 38 shared a common ancestor with strains from Japan (LC508802), Nigeria (MN606199), Germany (MF664194) and Italy (MN811085); and represented with them a separated clade with a bootstrap value of 99% as shown in Additional file 1: Figure S2.

### Discussion

The report found a novel strain of the 16S rRNA gene in tow isolates had not been previously reported. They shared a single nucleotide change from A to G distinguished them from the consensus sequence as a type of strain evolution. The Situation of mutant isolatesin separated branches makes it a most recent common ancestor of those groups. And revealed that the possible source of our mutant isolate is Ethiopia; this may be due that Ethiopia is the nearest country to Sudan among these countries. This agrees with similar outcomes found 16S rRNA sequencing can used to identify genetically atypical bacterial isolates from different sources [17, 26, 27].

The phylogenetic tree also exhibited different lineages of *S. aureus* strains detected from several hospitals in Sudan which indicated differential evolution. The isolates distributed in the three branches with those from Japan (LC508802), Nigeria (MN606199), Germany (MF664194), Italy (MN811085), Ethiopia (MK217496), Iraq (MN555444), Pakistan (MN611106), Bangladesh (MN611246), Egypt (MN556575), China (MN652637), India (MK165143) and USA (MF385261). The current results agree with previously reported study in Sudan by Mohamed et al. [28] that found genetic similarity in genomic sequence analyses of different *S. aureus* strains in the world in relation with isolated strains. Similar to the current study results, Raed et al*.* [29] indicated that genetic dimension between Iraq and the isolates of the world is extremely relative, and 16S rRNA analysis is considered a good discrimination approach for distinguishing unrelated isolates.

Today, some *S. aureus* strains are able to resist any known antibiotic and can cause serious infections in hospitals and the community [30]. Macrolides, Lincosamides and Streptogramines b antibiotics grouped into a single family because they share a similar binding site in subunit (23 s rRNA) of the bacterial ribosome [31–33]. Treatment of different infections with macrolide–lincosamide–streptogramin (MLS) antibiotics has led to resistance to these antibiotics via various mechanisms [30]. The mechanisms of resistance are mainly related to the inhibition of protein synthesis, this can be mediated by ribosomal binding site modification, active efflux mediated by *msr*A/B gene and enzymatic modification of antibiotics [32, 34]. Phenotypic expression of MLSB resistance in staphylococci can either inducible or constitutive which detected by D test [33].

From the short review above, we showed that the 12 (75%) iMLSB phenotype isolates prevailed over the 4 (25%) cMLSB phenotype which is slightly vary from other study performed in Sudan by Mahmoud et al. [35] which found (25.4%) of *S. aureus* isolates yielded inducible resistance. different results performed in Sudan by Makarem et al. [36] found all *S. aureus* isolates were sensitive to erythromycin antibiotic. It appears that there was no association between the occurrence of the resistant in MLSb *S. aureus* and 16S rRNA gene mutations. Overall this findings was in accordance with pervious findings found the amount of Methylated adenine in 16S rRNA is not affected by erythromycin [37].

Introduction of novel antimicrobials becoming a challenge to the microbiologist because of the emergence of new bacterial defense mechanisms like MLS b resistant and MRSA [38]. Using of essential oils like Atalantia sessiflora Guillaumin and galenic compound which has antibacterial and anti-inflammatory activity against multidrug-resistant strains of *S. aureus* were gave good results in many studies [39, 40] and its cytotoxicity effect was investigated on different cellular lines against both growing and stationary phase of *S. aureus* [41, 42]. Another studies discovered efflux inhibitory molecules and found the antibiotic action against resistant-*S. aureus* strains can inhibited by many compounds like NorA and P13CP which worked as efflux pump inhibitors [43, 44].

### Conclusion

The phylogenetic analysis of 16S rRNA sequences identified several lineages of *S. aureus* isolates detected from wound infections in Sudan. The mutation which discovered in 16S rRNA region do not associated with Macrolides, Lincosamides and Streptogramines b resistant phenotype. The application future direction of this paper is using DNA sequencing and insilico analysis depending on 16S rRNA and phylogeny approach to differentiate between closely related strains and study taxonomy relationships between bacteria. Future research should consider the potential effects of that mutation on resistance to other antibiotics.

## Limitations

However, the approach utilized suffers from the limitations that based on small sample size, an extra search is needed to highlight the variation which may occur and cover prevalent strains in different geographic regions in Sudan to obtain more complicated evolutionary events. Additionally, using (16S rRNA) as individual indication is regarding difficult to evaluate the bacterial diversity and identifying bacterial species [45], subsequently, the resolution of 16S rRNA that is extremely restricted with relative species but not quite characteristic to distinguish correlated staphylococcal spp. Finally, the highly conserved genomes may not have enough DNA polymorphisms in these restricted sequence proposed to display alleles [46].

## Supplementary Information

**Additional file 1: Table S1.** BLAST result of 16S rRNA gene: Sequencing ID in a gene bank, and compatibility of DNA sequences obtained from National Center Biotechnology Information (NCBI). **Figure: S1. (A)** Base pair substitution at position 249 from A to G which illustrated by arrows. Chromatograms edited using Finch TV software (B): Sequence alignment of 16S rRNA gene. **Figure S2.** Neighbour-joining and Maximum Parsimony trees based on the concatenated sequences of the 16S rRNA gene among *S. aureus *clinical isolates

## Data Availability

The datasets used and analysed during the current study available from the corresponding author on reasonable request.
