# Supplementary material for: First insights into molecular basis identification of 16 s ribosomal RNA gene of Staphylococcus aureus isolated from Sudan
Source: BMC Res Notes. 2021 Jun 25;14:240. doi: 10.1186/s13104-021-05569-w (PMC8229275; doi:10.1186/s13104-021-05569-w)

Additional file

**Table S1**: BLAST result of 16S rRNA gene: Sequencing ID in a gene bank, and compatibility of DNA sequences obtained from National Center Biotechnology Information (NCBI).

| **GenBank Accession Number** | **country** | **Source** | **Compatibility** |
| --- | --- | --- | --- |
| ID: LC_508802 | Japan | *Staphylococcus aureus* | 99% |
| ID: MN_611106 | Pakistan | *Staphylococcus aureus* | 99% |
| ID: MN_606199 | Nigeria | *Staphylococcus aureus* | 99% |
| ID: MN_611246 | Bangladesh | *Staphylococcus aureus* | 99% |
| ID_ MN556575 | Egypt | *Staphylococcus aureus* | 99% |
| ID_ MN555444 | Iraq | *Staphylococcus aureus* | 99% |
| ID_ MN652637 | China | *Staphylococcus aureus* | 99% |
| ID_ MF664194 | Germany | *Staphylococcus aureus* | 99% |

**Figure: S1. (A):** Base pair substitution at position 249 from A to G which illustrated by arrows. Chromatograms edited using Finch TV software (B): Sequence alignment of 16S rRNA gene

.**
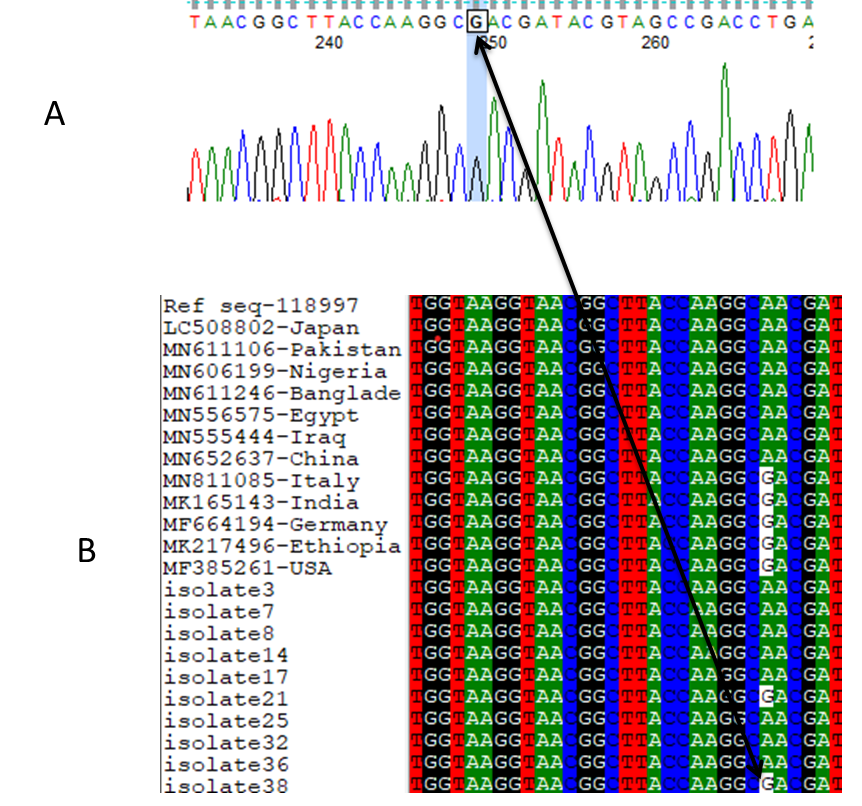
**

**Figure S2**: Neighbour-joining and Maximum Parsimony trees based on the concatenated sequences of the 16S rRNA gene among *S.aureus* clinical isolates


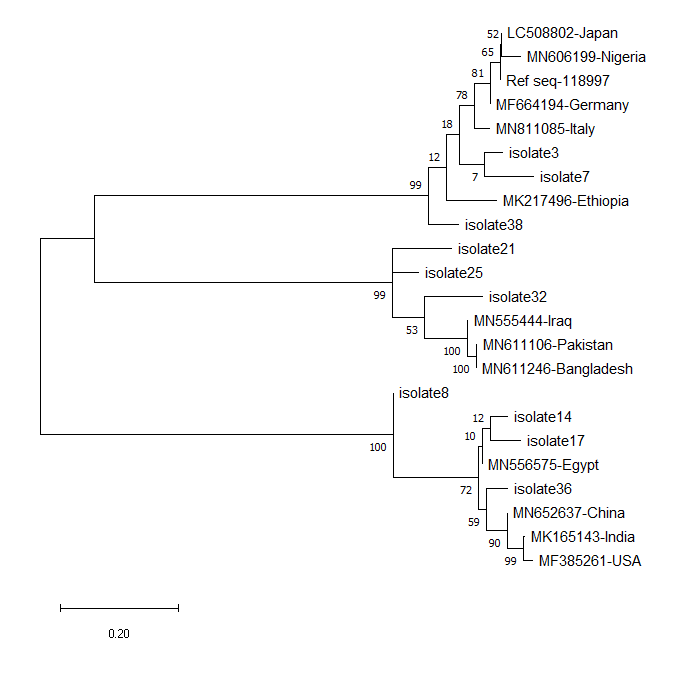

Supplement: Supplementary file 1 — Additional file 1: Table S1. BLAST result of 16S rRNA gene: Sequencing ID in a gene bank, and compatibility of DNA sequences obtained from National Center Biotechnology Information (NCBI). Figure: S1. (A) Base pair substitution at position 249 from A to G which illustrated by arrows. Chromatograms edited using Finch TV software (B): Sequence alignment of 16S rRNA gene. Figure S2. Neighbour-joining and Maximum Parsimony trees based on the concatenated sequences of the 16S rRNA gene among S. aureus clinical isolates [file 13104_2021_5569_MOESM1_ESM.docx]
